# Microspore Induced Doubled Haploids Production from Ethyl Methanesulfonate (EMS) Soaked Flower Buds Is an Efficient Strategy for Mutagenesis in Chinese Cabbage

**DOI:** 10.3389/fpls.2016.01780

**Published:** 2016-11-28

**Authors:** Yin Lu, Shuangyan Dai, Aixia Gu, Mengyang Liu, Yanhua Wang, Shuangxia Luo, Yujing Zhao, Shan Wang, Shuxin Xuan, Xueping Chen, Xiaofeng Li, Guusje Bonnema, Jianjun Zhao, Shuxing Shen

**Affiliations:** ^1^Key Laboratory of Vegetable Germplasm and Utilization of Hebei, Collaborative Innovation Center of Vegetable Industry in Hebei, College of Horticulture, Agricultural University of HebeiBaoding, China; ^2^Plant Breeding, Wageningen University & ResearchWageningen, Netherlands.

**Keywords:** *Brassica rapa*, EMS mutagenesis, microspore culture, Chinese cabbage, mutation

## Abstract

Chinese cabbage buds were soaked with Ethyl methanesulfonate (EMS) to induce mutagenesis. The influence of different EMS concentrations and treatment durations on microspore development, embryo production rate and seedling rate were evaluated in five Chinese cabbage genotypes. Mutations in four color-related genes were identified using high resolution melting (HRM) curves of their PCR products. The greatest embryo production and seedling rates were observed when buds were treated with 0.03 to 0.1% EMS for 5 to 10 min, while EMS concentrations greater than 0.1% were lethal to the microspores. In total, 142 mutants with distinct variations in leaf shape, leaf color, corolla size, flower color, bolting time and downy mildew resistance were identified from 475 microspore culture derived Doubled Haploids. Our results demonstrate that microspore derived Doubled Haploids from EMS soaked buds represents an efficient approach to rapidly generate homozygous Chinese cabbage mutants.

## Introduction

Naturally occurring mutations represent a major force driving evolution; however, they occur at an extremely slow rate. Artificially induced genetic variations represent important supplementary variation in plant breeding programs complementary to sources from natural origins (Konzak et al., 1976). Treating the seed or pollen of plants with the chemical mutagen EMS (Ethyl methanesulfonate) is one of the most commonly used approaches to generating large numbers of mutants. Favorable mutations can be identified by screening a mutagenesis library using high-throughput screening methods. EMS-induced mutagenesis is capable of establishing allelic mutations in genes via random point mutations that occur at a very high density, including non-sense, missense, splicing and cis-regulatory mutations (McCallum et al., 2000; Henikoff et al., 2004).

Targeting Induced Loci Lesions in Genomes (TILLING) is a reverse genetic approach that allows directed identification of mutations in specific genes. Since TILLING was originally developed in *Arabidopsis thaliana* in 2000 (McCallum et al., 2000), it has been widely used in genetic studies of agriculturally important species, including corn (Till et al., 2004), wheat (Chen et al., 2012), rice (Till et al., 2007), tomato (Minoia et al., 2010). High-performance liquid chromatography (HPLC) was initially used to identify gene mutations in TILLING populations, but that was costly and time-consuming. With the development of molecular biology and DNA sequencing technology, a number of novel methods has been developed for high-throughput genetic screening of mutagenesis libraries (Lochlainn et al., 2011). High resolution melting (HRM) analysis has recently emerged as a powerful tool for genotyping and screening TILLING populations at a low cost (Gady et al., 2009; Lochlainn et al., 2011; Zianni et al., 2013). Due to the use of heteroduplex-detecting, double-stranded DNA binding dyes rather than labeled primers, HRM can be used to efficiently identify genetic variations of PCR amplicons by measuring the dissociation temperature of double-stranded DNA (Martino et al., 2010).

Chinese cabbage (*Brassica rapa* L. ssp. *pekinensis*) is an important vegetable crop in Asia, with numerous uses in fresh and processed food products and with a high nutritional value (Zhao et al., 2005). Genetic modification and breeding programs are of critical importance for the development of a wide variety of specialized cultivars of Chinese cabbage for various markets. Nevertheless, Chinese cabbage breeding is challenging due to the narrow genetic backgrounds of the core collections. The available variations in the existing gene pool of Chinese cabbage must be increased to identify significant genes in key traits, such as disease/pest resistance, stress tolerance, nutritional quality, and plant architecture. A number of EMS-induced mutant populations have been created from several *Brassica* species. Two EMS-induced mutagenesis libraries were generated for the semi-winter *B. napus* doubled haploid line cv. Ningyou7 (Wang et al., 2008). The first EMS-mutated population of *B. rapa* was developed from the oil morphotype R-O-18 in the Department of Crop Genetics, John Innes Centre, Norwich of UK (Stephenson et al., 2010). In addition, Harloff et al. (2012) established two mutagenesis libraries of oilseed rape using EMS as a mutagen and identified sinapine biosynthesis mutants. The recently completed genome sequencing of Chinese cabbage provides access to a large number of gene sequences of this species in public databases, further encouraging the generation and screening of more comprehensive mutagenesis libraries (Wang et al., 2011).

The regeneration of doubled haploids from mutagenized donor plants has been used to increase the agronomic and biochemical variability of *Brassica*. Toward this goal, treatment of anthers and microspores with mutagens such as EMS and sodium azide is widely used to induce mutagenesis and to generate novel traits. For example, favorable variants of *B. napus* have been generated by treating microspores with EMS (Ferrie et al., 2008; Liu et al., 2010). A semi-dwarf mutant of *B. napus* was isolated from doubled haploid plants following EMS treatment of somatic embryos of microspore cultures (Liu et al., 2010). Mutants with a reduced height, altered fatty acid composition, and lower glucosinolate content were obtained by culturing microspores of the donor plants of Indian mustard (*B. juncea*) generated by treatment with EMS and ethyl nitrosourea (ENU; Prem et al., 2012). In the present study, five genotypes of Chinese cabbage were used to evaluate the effect of EMS on microspore development, embryo production rate, and seedling rate by soaking buds with different doses of EMS for different durations. Mutants were identified and characterized by HRM analysis. The results reveal an efficient method for the rapid production of homozygous mutants by combining chemical mutagenesis and microspore culture in Chinese cabbage.

## Materials and Methods

### Plant Materials and EMS Treatment

Five highly homozygous inbred or doubled haploid lines of Chinese cabbage (inbred line 85-1; doubled haploid lines 12-2, 12-7, A03 and A19) were grown in a greenhouse on an experimental farm at Hebei Agricultural University in Baoding, China in January 2012. After 2 months of vernalization at low temperatures (3–10°C), six seedlings of each genotype were transplanted in soil and cultivated normally.

Inflorescences of each genotype were collected after the first one to three flowers had opened. Flower buds ranging from 2.0 to 2.5 mm in length with middle uninucleate or early binucleate stage microspores were selected (Custers et al., 2001). Approximately 30 to 35 buds were soaked in solutions containing different concentrations of EMS (0.03, 0.05, 0.1, and 0.2%) at room temperature with gentle agitation. Buds were treated with each concentration of EMS for 5, 10 or 15 min. Buds soaked in sterile water were used as negative controls.

### Culture of Isolated Microspores

Microspores were cultured according to the protocol described by Custers et al. (2001). Buds treated with EMS were excised and surface-sterilized by dipping them in 70% ethanol for 30 s and then treating them with 2.5% NaClO for 5 min. Subsequently, they were rinsed with sterile distilled water three times for 5 min each. The buds were gently macerated in a tube containing 5 mL of B5 liquid medium, and microspores were collected by filtration through two layers of 45-μm nylon filter and then washed by centrifugation at 1000 r/min two times for 3 to 4 min each. After the final wash, the microspores were resuspended at a density of 1 × 10^5^ mL^-1^ in NLN13 medium containing active carbon at a concentration of 0.05 to 0.1 mg/mL. The suspension was poured into Petri dishes (60 mm diameter) for culturing (3 mL/dish). The cultures were incubated at 30°C for 24 h in the dark and then transferred to 25°C in the dark.

### Cytology of Microspore Embryogenesis

During microspore culturing, the embryonic development and regeneration were evaluated using an inverted microscope (Olympus CK40). Swollen cells were observed under a 10X ocular after exposure to a 24-h heat shock. Dividing cells and cell clusters were measured under a 10X ocular following incubation in the dark for 2 to 3 days. After an additional incubation in the dark for 7 to 14 days, the shapes of the embryo were examined under a 10X ocular. The total number of embryos for each genotype was calculated as the embryo production (average rate of embryo production = embryo number/buds number).

### Plant Regeneration

After 20 to 25 days of culture, embryos were counted for each genotype. The cotyledonary embryos were gradually introduced to light over 48 to 72 h. Next, embryos with green cotyledons were rooted on 1/2 MS medium. The cultures were grown in a growth chamber and maintained at 25°C using a photoperiod of 14 h light and 10 h darkness. Eight weeks after the embryos were transferred to regeneration medium, plantlets with 5 to 6 true leaves were transplanted to soil in the greenhouse. The seedling rate of the different genotypes was counted (average seedling rate = plantlet number/embryo number).

### Phenotypic Investigation and Ploidy Level Identification

Compared with wild type (at least 10 plants of each wild type), phenotypic variations including leaf shape, leaf color, corolla size, flower color, bolting resistance during the vegetative growth period (seedling, rosette, heading and harvest stage) and reproductive period were investigated. Leaf shape was recorded as long ellipse, long inverted ellipse, broad ellipse, close round and round at vegetative stage. Leaf color was scored by eye as green, yellow-green, light yellow and light white at rosette, heading and reproductive stage. At reproductive stage, flower color was scored by eye as white and yellowish color, in which at least 10 flowers of each plant were phenotyped. Corolla size was measured by ruler and compared with wild types. Bolting time was recorded when the first flower bud appeared. Downy mildew tolerance was measured after touch inoculation by contaminated leaves. The downy mildew symptoms were evaluated when 2–3 leaves were contaminated.

Ploidy levels (diploid, haploid and tetraploid) were identified by flow cytometry.

### PCR Analysis for HRM Analysis

Sixteen primers were designed using Primer 5 to amplify 100 to 400 bp regions within four genes related to the flower color pathway (Supplementary Table 1). Dihydroflavonol 4-Reductase (DFR) and chalcone isomerase (CHI) were both key enzymes of the flavonoid biosynthesis pathway (Nishihara et al., 2005). They play impartment roles in pigment accumulation of the seed coat and flower. The over-expression of DFR and CHI make flowers color darker (Aida et al., 2000; Nishihara et al., 2005). Genomic DNA was extracted from fresh leaves of mutants and wild type plants (85-1) according to the procedure described by Beek et al. (1992). For the PCR analysis, 10-μL reaction volumes were used: 2 μL of 5X PCR buffer, 0.8 μL of 2.5 mM of dNTP, 0.5 μL of 2.5 mM of both forward and reverse primers, 1 μL of 1X LC green Plus, 0.1 μL of 5U of Taq enzyme, 1 μL of 50 ng of genomic DNA, and 4.5 μL of double-distilled water. The PCR reactions were performed using a 7300 Thermocycler (Bio-Rad, USA) with the following conditions: 98°C for 30 s, 35 cycles at 98°C for 10 s, annealing at 60°C for 10 s, and an extension at 72°C for 30 s, followed by an additional denaturation step at 94°C for 30 s and a cool down to 25°C for 30 s to facilitate heteroduplex formation.

### Analysis of HRM Products Using the Lightscanner^TM^ System

Following PCR, each sample was transferred to a 96-well LightScanner System (Idaho Technology Inc., USA) and overlaid with mineral oil. The samples were heated from 70°C up to 96°C using a ramp rate of 0.10°C s^-1^. LightScanner software (Version 2.0) was used for data analysis according to Montgomery et al. (2007). A saturating DNA binding dye was introduced during DNA amplification, enabling the differentiation of PCR products based on their dissociation behavior as the temperature increased. The melting profiles were calibrated using internal oligonucleotide controls. They were subsequently normalized, temperature-shifted, grouped, and displayed as fluorescence versus temperature plots (Montgomery et al., 2007).

### Confirmation of Mutations by PCR and DNA Sequencing

The genes were amplified from genomic DNA of the HRM-identified mutants and wild type plants (85-1) using on one side primers used for HRM screening, combined with opposite strand primers to obtain a fragment of approximately 800 bp (Supplementary Table 1). The PCR products were then sent to Sangon Biotech Co., Ltd in Shanghai of China to be sequenced with three replications. The sequences from wild-type and mutant samples were aligned using DNAMAN software to identify mutations.

## Results

### Impact of EMS on Microspore Embryogenesis

**FIGURE 1:**
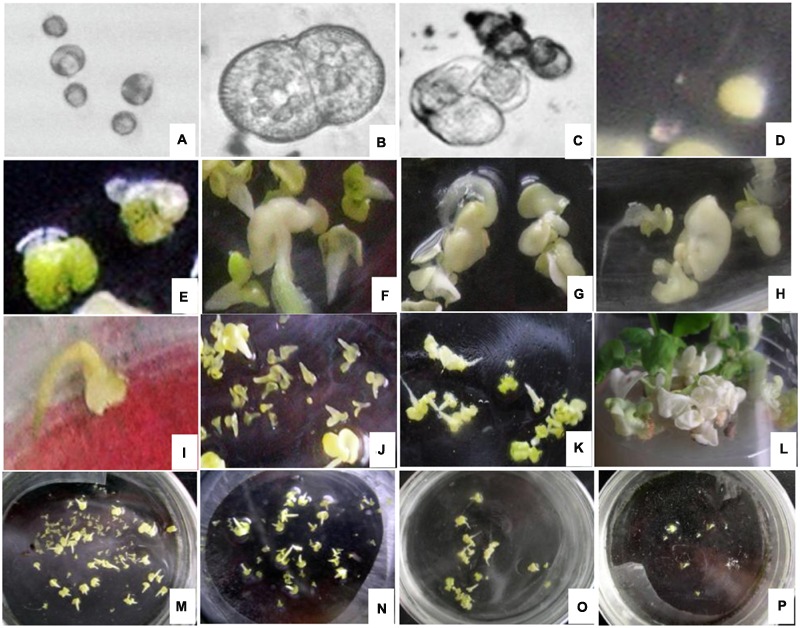
**Cytology of microspore embryogenesis with and without EMS treatment (A)** Swollen cell observed after heat shock for 24 h. **(B)** Dividing cells after incubation in the dark for 2 to 3 days. **(C)** Cell cluster. **(D)** Globular embryo observed after incubation in the dark for 7 to 14 days. **(E)** Heart-shaped embryo. **(F)** Cotyledon embryos. **(G,H)** Deformed embryo. **(I)** brown and died embryo. **(J)** Lagging embryo development. **(K)** Albino embryo. **(L)** Albino seedlings. **(M)** Embryo production without EMS. **(N)** Embryonic development with 0.03% EMS treatment. **(O)** Embryonic development after 0.05% EMS treatment. **(P)** Embryonic development after 0.1% EMS treatment. **(A–F,M)** without EMS; **(G–L** and **N–P)** with EMS.

The cytology of microspore embryogenesis is illustrated in **Figure 1**. After the 24 h heat treatment in NLN-13 medium, the majority of the microspores that were not treated with EMS began to swell, increasing 1- to 2-fold in diameter (**Figure 1A**). After culturing in the dark for 2 to 3 days, the swollen microspores initiated the first division (**Figure 1B**). Cell clusters (**Figure 1C**) appeared after 5 days of culture. After 7 to 14 days, visible embryos with different shapes (spheres, **Figure 1D**; hearts, **Figure 1E**; deformed embryos, **Figures 1G,H**) were observed. Cotyledon embryos (**Figure 1F**) appeared after 16 to 20 days of culture.

In general, embryo development was delayed after EMS treatment and spherical embryo’s were observed after 10 days of culture with slow growth. Development of most globular embryos was halted (**Figure 1J**), however, small cotyledonal embryos appeared following 24 to 26 days of culture. When cultured in the light, some of the cotyledonal embryos did not become green but turned brown and died (**Figure 1I**), producing albino seedlings (**Figures 1K,L**). With increasing EMS concentration and duration of exposure in all genotypes, the number of embryos produced clearly decreased (**Figures 1M–P**).

### Impact of EMS on the Rate of Microspore Development in Embryos

Treatment with EMS significantly affected embryo production in all of the tested genotypes (**Table 1**, Supplementary Tables 2 and 3). At an EMS concentration ranging from 0.03 to 0.10%, the rate of embryo production in the various genotypes differed with different treatment durations. At high concentrations (>0.2%) of EMS or long durations (>10 min) of treatment, microspores were no more viable. When the concentration reached 0.2%, no embryos were detected for any of the tested genotypes; a few embryos were observed at treatment durations longer than 10 min.

**Table 1:** Average rates of embryo production from the microspore embryoids of five Chinese cabbage genotypes treated with different concentrations of EMS for different durations.

EMS concentration (%)/time (min)	Average rate of embryo production (embryos per bud)				
	85-1	12-2	12-7	A03	A19
0.00/5	13.67	–	–	13.31	–
0.00/10	15.69	–	–	12.52	–
0.00/15	14.13	–	–	11.39	–
0.03/5	7.28	1.77	0.63	3.03	4.11
0.03/10	5.61	0.00	0.13	0.35	0.20
0.05/5	–	1.89	0.09	16	12.2
0.05/10	–	0.08	0.00	0.00	0.42
0.05/15	–	0.00	0.00	0.13	0.00
0.10/5	5.81	1.83	1.61	5.22	1.64
0.10/10	4.83	0.40	0.32	0.00	0.00
0.10/15	0.46	0.00	0.00	0.00	0.00
0.20/5	0.00	0.00	0.00	0.00	0.00
0.20/10	0.00	0.00	0.00	0.00	0.00
0.20/15	0.00	0.00	0.00	0.00	0.00

*‘–’ means no data.*

### Impact of EMS on the Seedling Rate of Microspores

The seedling rate of all five genotypes differed depending of the different EMS treatments (**Table 2**, Supplementary Tables 4 and 5). In addition there was a clear genotypic effect, as the five genotypes had different seedling rates. With increasing EMS concentration and treatment duration, survival of seedlings decreased. At high concentrations (>0.2%) or long durations (>10 min) of EMS treatment, almost no embryos developed into seedlings.

**Table 2:** Average seedling rate of five Chinese cabbage genotypes treated with different concentrations of EMS for different durations.

EMS concentration (%)/time (min)	Average seedling rate				
	85-1	12-2	12-7	A03	A19
0.00/5	80.25	–	–	75.62	–
0.00/10	79.60	–	–	77.53	–
0.00/15	83.51	–	–	81.57	–
0.03/5	31.53	93.00	54.40	28.10	7.01
0.03/10	30.11	0.00	40.00	7.01	0.00
0.05/5	–	7.13	76.23	15.46	0.00
0.05/10	–	3.68	0.00	0.00	33.33
0.05/15	–	0.00	0.00	0.00	0.00
0.10/5	30.97	45.38	68.42	13.13	0.00
0.10/10	75.41	2.25	4.97	0.00	0.00
0.10/15	17.50	0.00	0.00	0.00	0.00
0.20/5	0.00	0.00	0.00	0.00	0.00
0.20/10	0.00	0.00	0.00	0.00	0.00
0.20/15	0.00	0.00	0.00	0.00	0.00

*‘–’ means no data.*

Considering the rates of embryo production and plant generation, the optimum concentration and duration of EMS treatment of buds to generate mutants from microspore cultures was 0.10% for 5 min.

### Phenotypic Variations of Mutants Induced by EMS

**FIGURE 2:**
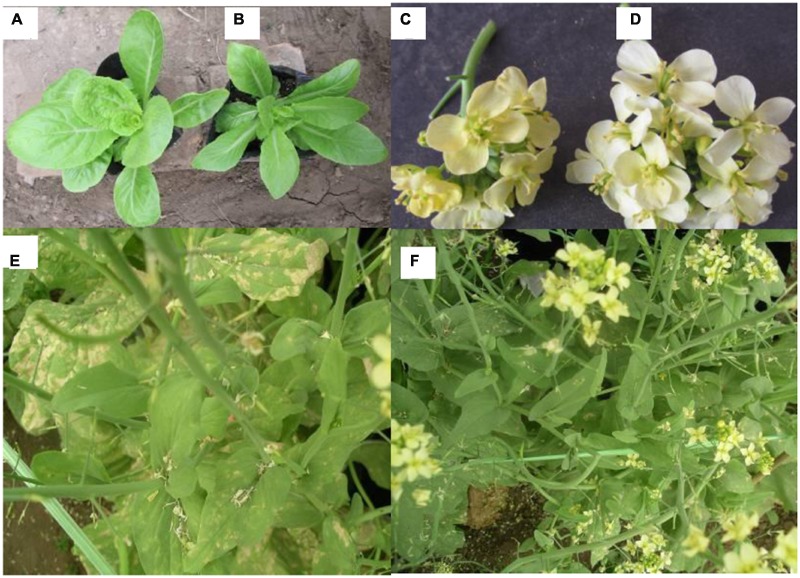
**Phenotypic changes of mutants in the M_1_ generation of the Chinese cabbage after soaking buds with EMS (A)** Wild type A03. **(B)** A03 leaf shape variation mutant (blade tip of mutants was slightly pointed). **(C)** 85-1 flower color variation mutant (yellowish color). **(D,E)** Wild type 85-1. **(F)** 85-1 tolerance to downy mildew mutant.

In total, 475 regenerated plants were obtained from EMS treated buds of the five genotypes (0.10% for 5 min). Some typical phenotypic variations among the 142 diploid mutants obtained by EMS treatment are shown in **Figure 2**. Plants with leaf variation (**Figure 2B**; 68 leaf shape mutants, 40 leaf color mutants, four leaf edge shape mutants), 15 plants with flower color variation (**Figure 2D**), one plant with tolerance to downy mildew (**Figure 2F**), six plants with corolla size variation and eight plants with bolting time variation were observed. Leaf shape of the wild type ‘A03’ was ellipse, while the blade tip of mutants was slightly pointed; flower color was changed from white to yellowish.

### Genotyping of TILLING Population Using HRM

To demonstrate that HRM is suitable for efficient mutant identification in the TILLING lines, four target genes related to flower color formation were selected for the HRM analyses (DFR1 Bra027457, DFR2 Bra010535, CHI1 Bra009101 and CHI 2 Bra037180; Supplementary Table 1).

**FIGURE 3:**
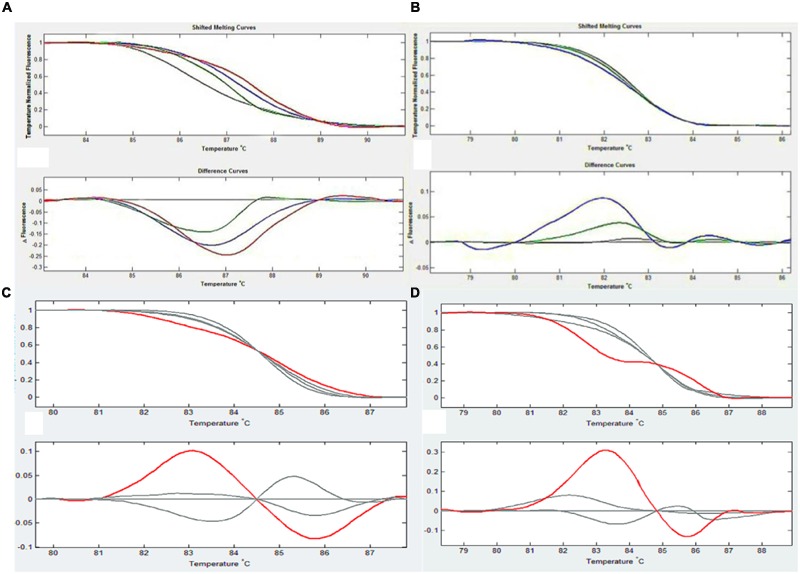
**An example of HRM analysis in each figure, the upper curve reflects the difference in dissolution curves after removing differences among the different holes, and the lower curve shows the changes in fluorescence that occurred during the process of dissolution.** The default value is the change in wild type (gray line), which allows the detection of changes in mutant (colored lines) and wild type plants at the same fluorescence level. **(A)** CHI1 test results. The red line is 11-6, the blue line is 11-8 and the green line is 11-18. **(B)** DFR2 test results. The blue line is 11-6 and the green is 11-8, the color is different for different point mutations. **(C)** CHI2 test results. The red line is 13-8. **(D)** DFR1 test results. The red line is 11-17.

HRM analysis revealed individual microspore derived plants with mutations in any of these four genes (**Figure 3**). Seven out of the 142 plants were considered to possess mutations in any of the four flower color genes, including five diploid mutants, one haploid mutant and one tetraploid plant.

### Confirmation of Mutations by DNA Sequencing

PCR amplification of the putative mutant genes and DNA sequencing were used to confirm the sequence variations of the five diploid mutants identified by HRM (**Table 3**). Compared to wild type, all of the five diploid mutant plants possessed mutations, in the sequenced genes including six single nucleotide polymorphisms, one deletion (one base) and one insertion mutant. Four of these diploid mutant plants displayed changes in flower color, among which three (11-8, 11-17, 11-18) turned yellow at the late stage and one (11-6) was yellow at all growth stages, confirming these mutations.

**Table 3:** Individual mutants identified by HRM that were confirmed by DNA sequencing.

Mutant individual	Gene name	Seq length	Gene Mutation	Mutation position (bp from initiation codon)	Mutation Location (amino-acid)	Mutation type	Amino-acid Mutation	Color variation
11-6	*CHI 1*	988	A deletion	400	–	Non-coding	–	color yellowish
	*DFR2*	1408	G to A	623	137	Missense	Glu to Lys (E to K)	
			A insertion	657	–	Non-coding	–	
11-8	*CHI 1*	988	C to T	193	40	Missense	Ala to Val (A to V)	yellowish color in late stage
11-18	*CHI 1*	988	G to A	880	171	silent	–	yellowish color in late stage
	*DFR2*	1408	G to A	623	137	Missense	Glu to Lys (E to K)	
13-8	*CHI 2*	1131	G to A	634	–	Non-coding	–	same as wild type
11-17	*DFR1*	1558	G to A	1552	385	Missense	Ala to Thr (A to T)	yellowish color in late stage

## Discussion

In investigations of gene function, the identification of plants with mutations in these genes is fundamental. Here, we described the rapid generation of mutants by microsporogenesis of EMS treated flower buds. Our results demonstrate that high-throughput identification of mutations in specific genes using HRM is suitable for reverse genetic studies of Chinese cabbage. The recently completed genome sequence of *B. rapa* significantly impacts genetic studies of this species as comparative genomics and the design of species-specific primers have become relatively straightforward practices (Wang et al., 2011).

In most of the published EMS-induced mutagenesis studies, mutants were obtained by treating seeds with EMS. Because seeds have a high tolerance to mutagens, the suitable EMS concentration and duration of treatment for seeds are higher than those for microspores. For *B. napus* mutagenesis, an EMS concentration of 0.3 to 0.6% and a treatment duration for 12 to 18 h are the best conditions for obtaining a large number of mutants while maintaining sufficient vigorous, fertile plants (Wang et al., 2008; Harloff et al., 2012). Ferrie et al. (2008) developed a microspore mutagenesis protocol for the production of double haploid *Brassica* lines with novel fatty acid profiles in seed oil. The isolated microspores were first cultured in EMS for 1.5 h. Next, the EMS was removed and normal microspore culture was performed. This protocol has been previously used to generate over 80000 *Brassica* haploid/doubled haploid plants (Ferrie et al., 2008).

In the present study, we performed bud mutagenesis using buds treated with different concentrations and durations of EMS followed by the culture of isolated microspores from five Chinese cabbage genotypes. Compared with treating seeds with EMS, homozygous mutants can be obtained quickly by treating buds combined with microspore culture. Based on the results for the microspore embryogenesis rate, seedling rate, and the number of effective mutants, the appropriate EMS concentration for mutagenesis ranged from 0.03 to 0.1%, which is lower than that used to treat *B. napus* microspores (0.1 to 0.2%, as reported by Ferrie et al., 2008). In addition, the optimal duration of EMS treatment ranged from 5 to 10 min, which is much shorter than the 1.5 h of culture used for *B. napus* microspores (Ferrie et al., 2008). In another report published by Xie (2008), *B. napus* mutagenesis was performed by treating microspores with 0.04 to 0.08% EMS for 10 to 20 min. The differences observed between *B. rapa* and *B. napus* may be due their genotypes or the physiological stage of the plants from which the buds were harvested. Our results demonstrate that EMS treatment of microspores is an efficient procedure to generate mutations resulting in highly diverse phenotypes of Chinese cabbage. The mutation ratio is very high (29.89%; 142 phenotypic mutant plants out of a total of 475 plants; Supplementary Table 4). This high percentage possible biased, as from the 1257 plantlets (from 3844 microspore cultured embryos from buds treated with 0.1% EMS), this subset of 475 was selected before acclimatization and field-planting.

Using HRM screening, five mutants with flower color were obtained from the microspore culture plantlets derived from EMS bud mutagenesis. In combination with validation of mutants by direct sequencing of PCR fragments, HRM was shown to be useful for EMS mutant screening as a low cost, easy to conduct, high throughput method. The traditional platform of Conformation Sensitive Capillary Electrophoresis endonuclease-Li-Cor (Triques et al., 2007), despite being more sensitive than HRM in pooled samples in terms of heteroduplex detection, is labor intensive due to the numerous steps involved in the multiple procedures (PCR, enzymatic reactions, gel preparation and loading and visual analysis; Gady et al., 2009). PCR-RFLP has also been used to analyze allelic series of mutants. This procedure, which identifies mutations that cause changes in a restriction site, is not sensitive enough to identify mutations that do not modify the restriction profile (Monis et al., 2005). TaqMan assays could also be used to identify point mutants, but this technique requires expensive probes and assay optimization (Parsons and Heflich, 1997). Next generation sequencing (NGS) recently has been used to successfully identify rare mutations in a mutant population of *B. napus* (Gilchrist et al., 2013). In *B. rapa*, paralogous genes are common and mutations of both copies of these genes may be required to generate a phenotype. When screening mutagenesis libraries, it is extremely important to request that high-throughput genotyping of multiple alleles of many genes be performed quickly and efficiently. In our previous studies (Lu, 2014; Lu et al., 2014), one Chinese cabbage A03 mutant library contained 4253 M_1_ families were constructed by EMS seed treatment in combination with bud treatment with EMS. Five available specific primers were designed for the one gene *Bra027829* (related to auxin) and used to screen pools of DNA samples from 8 plants using HRM technology, resulting in 83 point mutations. These results demonstrate that HRM is also a high-throughput technique for screening larger mutant libraries induced by EMS.

In summary, in the present study, we provide evidence that microsporogenesis from buds of Chinese cabbage soaked in an appropriate concentration of EMS is an efficient way to generate mutantions which can be identified by high-throughput HRM screening.

## Ethics Statement

The study was approved by Hebei Agricultural University, China. All provided written informed consent.

## Author Contributions

YL got mutants by EMS treatment and contributed to writing; SD and AG did microspore culture and help in writing; ML, YW, SL, YZ, and SW did HRM and PCR, and helped in the discussion and drafted the manuscript; SX, XC, and XL did field experiment and performed the analysis; GB helped writing and language editing; JZ and SS designed the study and supervised the whole experiment and help in writing.

## Conflict of Interest Statement

The authors declare that the research was conducted in the absence of any commercial or financial relationships that could be construed as a potential conflict of interest.
