# Supplementary material for: Microspore Induced Doubled Haploids Production from Ethyl Methanesulfonate (EMS) Soaked Flower Buds Is an Efficient Strategy for Mutagenesis in Chinese Cabbage
Source: Front Plant Sci. 2016 Nov 28;7:1780. doi: 10.3389/fpls.2016.01780 (PMC5147456; doi:10.3389/fpls.2016.01780)
Supplement: Supplementary file 1 [file Data_Sheet_1.doc]

**SUPPLEMENTAL TABLE 1｜Primers used to amplify the four color-related genes in the HRM analysis.**

| **Gene name** | **Locus name *B. rapa*** | **Primer**  **name** | **Forward sequence (5’–>3’)** | **Reverse sequence (5’–>3’)** |
| --- | --- | --- | --- | --- |
| *DFR1* | Bra027457 | DFR1-1 | CTAAGCACAGATCTGCTGTGCCG | TATGAACTCTTTGGAACAGGTTTG |
|  |  | DFR1-2 | TCAAACCTGTTCCAAAGAGT | CTATGCCGCCTAGCCTTATTACCGC |
|  |  | DFR1-3 | AGAGAGCGCGGTAATAAGGCTAGGC | AATGAAAGCATGTGATAAGGCAAAG |
|  |  | DFR1-4 | GTCAAGATCACTCCAATCGTTTTCA | GTTTTCCACATAGCAACTCCC |
|  |  | DFR1-5 | ATGGGAGTTGCTATGTGG | ATGGTAGCTCACAAAGAGACCGTG |
| *DFR2* | Bra010535 | DFR2-1 | TGGATCAAACCAAAGGAAAAG | TGAGGACAGGAGAAGCAGTATG |
|  |  | DFR2-2 | GGAGTTTTCCATACTGCTTT | GCAGAGTTCCACAGAGGTCC |
|  |  | DFR2-3 | GGACCTCTGTGGAACTCTGC | ATCTGTCCATGCCACTGAAA |
|  |  | DFR2-4 | ATGGGTTATATTCATATAGACGAC | TTAAGAAACTACATGAGATAAAT |
| *CHI1* | Bra009101 | CHI1-1 | TAGGTTAAAACTGCCGACA | TTGGCTGCTGAGGATAAG |
|  |  | CHI1-2 | TTCTTCTTCCTCCTCGTA | CTCCGGTAAACTTAATCAGTCC |
|  |  | CHI1-3 | GAAATCACAAAGCCAAAAC | GTCATGGTTCACGAGGTT |
| *CHI2* | Bra037180 | CHI2-1 | TCATTCCTCGTCGTCTTC | TCAACGCAAGTACCAAAG |
|  |  | CHI2-2 | CTTTGGTACTTGCGTTGA | GGACTTCTCGCTCCGACT |
|  |  | CHI2-3 | CGGAGTCGGAGCGAGAAGTCCCATC | AGAGAAACCAGAGGAGTCGAAATCA |
|  |  | CHI2-4 | CTCTTCCCTACAACAGCC | AGAGACACCATCATCAACG |

| **SUPPLEMENTAL TABLE 2｜The number of buds treated and embryoids obtained by isolated microspore culture of five Chinese cabbage genotypes treated with different concentrations of EMS for different durations** | | | | | | | | | | | | | | | | | | |  |
| --- | --- | --- | --- | --- | --- | --- | --- | --- | --- | --- | --- | --- | --- | --- | --- | --- | --- | --- | --- |
| **EMS concentration (%)/time (min)** | **85-1** | | |  | **12-2** | | |  | **12-7** | | |  | **A03** | | |  | **A19** | | |
|  | **Embryoid number** | **Bud number** | **Dish**  **number** |  | **Embryoid number** | **Bud number** | **Dish**  **number** |  | **Embryoid number** | **Bud number** | **Dish**  **number** |  | **Embryoid number** | **Bud number** | **Dish**  **number** |  | **Embryoid number** | **Bud number** | **Dish**  **number** |
| 0.00/5 | 2051 | 150 | 5 |  | － | － | － |  | － | － | － |  | 1997 | 150 | 5 |  | － | － | － |
| 0.00/10 | 2354 | 150 | 5 |  | － | － | － |  | － | － | － |  | 1878 | 150 | 5 |  | － | － | － |
| 0.00/15 | 2120 | 150 | 5 |  | － | － | － |  | － | － | － |  | 1708 | 150 | 5 |  | － | － | － |
| 0.03/5 | 1158 | 159 | 5 |  | 216 | 122 | 4 |  | 132 | 209 | 6 |  | 475 | 157 | 5 |  | 674 | 164 | 5 |
| 0.03/10 | 931 | 166 | 5 |  | 0 | 167 | 5 |  | 24 | 188 | 6 |  | 63 | 181 | 6 |  | 35 | 175 | 5 |
| 0.05/5 | － | － | － |  | 421 | 223 | 7 |  | 152 | 1689 | 50 |  | 2480 | 155 | 5 |  | 1866 | 153 | 5 |
| 0.05/10 | － | － | － |  | 136 | 1700 | 50 |  | 0 | 162 | 5 |  | 0 | 165 | 5 |  | 71 | 168 | 5 |
| 0.05/15 | － | － | － |  | 0 | 164 | 5 |  | 0 | 154 | 5 |  | 20 | 153 | 5 |  | 0 | 166 | 5 |
| 0.10/5 | 1401 | 241 | 7 |  | 435 | 238 | 7 |  | 324 | 201 | 6 |  | 1080 | 207 | 6 |  | 256 | 156 | 5 |
| 0.10/10 | 932 | 193 | 6 |  | 92 | 230 | 7 |  | 165 | 515 | 35 |  | 0 | 172 | 5 |  | 0 | 155 | 5 |
| 0.10/15 | 92 | 199 | 6 |  | 0 | 156 | 5 |  | 0 | 156 | 5 |  | 0 | 180 | 6 |  | 0 | 173 | 5 |
| 0.20/5 | 0 | 161 | 5 |  | 0 | 168 | 5 |  | 0 | 152 | 5 |  | 0 | 189 | 6 |  | 0 | 156 | 5 |
| 0.20/10 | 0 | 150 | 5 |  | 0 | 171 | 5 |  | 0 | 155 | 5 |  | 0 | 210 | 7 |  | 0 | 161 | 5 |
| 0.20/15 | 0 | 155 | 5 |  | 0 | 158 | 5 |  | 0 | 152 | 5 |  | 0 | 181 | 6 |  | 0 | 161 | 5 |

‘－’means no data.

**SUPPLEMENTAL TABLE 3｜Significant test for Impact of EMS on the Rate of Microspore Development in Embryos**

**A Significant test for different time and genotypes under same EMS concentration treatment**

| Genotypes   | EMS concentration(%) / time (min) | Mean | standard deviation | 5% significant level | 1% significant level |  | | --- | --- | --- | --- | --- | --- | | 85-1 | 0.00/5 | 13.673 | 0.731 | ab | AB | |  | 0.00/10 | 15.693 | 0.700 | a | A | |  | 0.00/15 | 14.133 | 1.240 | ab | AB | | A03 | 0.00/5 | 13.313 | 1.412 | bc | AB | |  | 0.00/10 | 12.520 | 0.360 | bc | B | |  | 0.00/15 | 11.387 | 1.592 | c | B |  | Genotypes | EMS concentration(%) / time (min) | | Mean | | standard deviation | | 5% significant level | | 1% significant level | | | --- | --- | --- | --- | --- | --- | --- | --- | --- | --- | --- | | 85-1 | 0.03/5 | | 7.283 | | 0.394 | | a | | A | | |  | 0.03/10 | | 5.610 | | 0.252 | | b | | B | | | 12-2 | 0.03/5 | | 1.773 | | 0.242 | | e | | E | | |  | 0.03/10 | | 0.000 | | 0.000 | | h | | G | | | 12-7 | 0.03/5 | | 0.630 | | 0.035 | | f | | F | | |  | 0.03/10 | | 0.123 | | 0.023 | | gh | | G | | | A03 | 0.03/5 | | 3.023 | | 0.137 | | d | | D | | |  | 0.03/10 | | 0.347 | | 0.035 | | fg | | FG | | | A19 | 0.03/5 | | 4.110 | | 0.062 | | c | | C | | |  | 0.03/10 | | 0.197 | | 0.025 | | gh | | G | | | Genotypes | | EMS concentration(%) / time (min) | | Mean | | standard deviation | | 5% significant level | | 1% significant level | | 12-2 | | 0.05/5 | | 1.8867 | | 0.0635 | | c | | C | |  | | 0.05/10 | | 0.080 | | 0.000 | | d | | D | |  | | 0.05/15 | | 0.000 | | 0.000 | | d | | D | | 12-7 | | 0.05/5 | | 0.090 | | 0.000 | | d | | D | |  | | 0.05/10 | | 0.000 | | 0.000 | | d | | D | |  | | 0.05/15 | | 0.000 | | 0.000 | | d | | D | | A03 | | 0.05/5 | | 16.0067 | | 1.0538 | | a | | A | |  | | 0.05/10 | | 0.000 | | 0.000 | | d | | D | |  | | 0.05/15 | | 0.000 | | 0.000 | | d | | D | | A19 | | 0.05/5 | | 12.1967 | | 0.3729 | | b | | B | |  | | 0.05/10 | | 0.4267 | | 0.0808 | | d | | D | |  | | 0.05/15 | | 0.000 | | 0.000 | | d | | D |  | Genotypes | EMS concentration(%) / time (min) | Mean | standard deviation | 5% significant level | 1% significant level | | --- | --- | --- | --- | --- | --- | | 85-1 | 0.1/5 | 5.8133 | 0.2031 | a | A | |  | 0.1/10 | 4.8267 | 0.132 | c | C | |  | 0.1/15 | 0.4633 | 0.0808 | e | E | | 12-2 | 0.1/5 | 1.8267 | 0.1137 | d | D | |  | 0.1/10 | 0.400 | 0.0656 | e | E | |  | 0.1/15 | 0.000 | 0.000 | f | F | | 12-7 | 0.1/5 | 1.6133 | 0.1305 | d | D | |  | 0.1/10 | 0.320 | 0.000 | e | EF | |  | 0.1/15 | 0.000 | 0.000 | f | F | | A03 | 0.1/5 | 5.22 | 0.3195 | b | B | |  | 0.1/10 | 0.000 | 0.000 | f | F | |  | 0.1/15 | 0.000 | 0.000 | f | F | | A19 | 0.1/5 | 1.640 | 0.3451 | d | D |   **B Significant test for different concentration and genotypes under same time treatment**   | Genotypes | EMS concentration(%) / time (min) | Mean | standard deviation | 5% significant level | 1% significant level | | --- | --- | --- | --- | --- | --- | | 85-1 | 0.00/5 | 13.673 | 0.731 | b | B | |  | 0.03/5 | 7.2833 | 0.3937 | d | D | |  | 0.1/5 | 5.8133 | 0.2031 | e | E | |  | 0.2/5 | 0.0000 | 0.0000 | i | J | | 12-2 | 0.03/5 | 1.7733 | 0.2421 | h | H | |  | 0.05/5 | 1.8867 | 0.0635 | h | H | |  | 0.1/5 | 1.8267 | 0.1137 | h | H | |  | 0.2/5 | 0.0000 | 0.0000 | i | J | | 12-7 | 0.03/5 | 0.6300 | 0.0346 | i | IJ | |  | 0.05/5 | 0.0900 | 0.0000 | i | J | |  | 0.1/5 | 1.6133 | 0.1305 | h | HI | |  | 0.2/5 | 0.0000 | 0.0000 | i | J | | A03 | 0.00/5 | 13.3133 | 1.4121 | b | B | |  | 0.03/5 | 3.0233 | 0.1365 | g | G | |  | 0.05/5 | 16.0067 | 1.0538 | a | A | |  | 0.1/5 | 5.2200 | 0.3195 | e | E | |  | 0.2/5 | 0.0000 | 0.0000 | i | J | | A19 | 0.03/5 | 4.1100 | 0.0624 | f | F | |  | 0.05/5 | 12.1967 | 0.3729 | c | C | |  | 0.1/5 | 1.6400 | 0.3451 | h | HI | |  | 0.2/5 | 0.0000 | 0.0000 | i | J |  | Genotypes | EMS concentration(%) / time (min) | Mean | standard deviation | 5% significant level | 1% significant level | | --- | --- | --- | --- | --- | --- | | 85-1 | 0.00/15 | 14.133 | 1.240 | a | A | |  | 0.1/15 | 0.463 | 0.081 | c | C | |  | 0.2/15 | 0.000 | 0.000 | c | C | | 12-2 | 0.05/15 | 0.000 | 0.000 | c | C | |  | 0.1/15 | 0.000 | 0.000 | c | C | |  | 0.2/15 | 0.000 | 0.000 | c | C | | 12-7 | 0.05/15 | 0.000 | 0.000 | c | C | |  | 0.1/15 | 0.000 | 0.000 | c | C | |  | 0.2/15 | 0.000 | 0.000 | c | C | | A03 | 0.00/15 | 11.387 | 1.592 | b | B | |  | 0.05/15 | 0.000 | 0.000 | c | C | |  | 0.1/15 | 0.000 | 0.000 | c | C | |  | 0.2/15 | 0.000 | 0.000 | c | C | | A19 | 0.05/15 | 0.000 | 0.000 | c | C | |  | 0.1/15 | 0.000 | 0.000 | c | C | |  | 0.2/15 | 0.000 | 0.000 | c | C |  | Genotypes | EMS concentratiion(%) / time (min) | Mean | standard deviation | 5% significant level | 1% significant level | | --- | --- | --- | --- | --- | --- | | 85-1 | 0.00/10 | 15.693 | 0.700 | a | A | |  | 0.03/10 | 5.610 | 0.252 | c | C | |  | 0.1/10 | 4.827 | 0.132 | d | D | |  | 0.2/10 | 0.000 | 0.000 | f | E | | 12-2 | 0.03/10 | 0.000 | 0.000 | f | E | |  | 0.05/10 | 0.080 | 0.000 | ef | E | |  | 0.1/10 | 0.400 | 0.066 | e | E | |  | 0.2/10 | 0.000 | 0.000 | f | E | | 12-7 | 0.03/10 | 0.123 | 0.023 | ef | E | |  | 0.05/10 | 0.000 | 0.000 | f | E | |  | 0.1/10 | 0.320 | 0.000 | ef | E | |  | 0.2/10 | 0.000 | 0.000 | f | E | | A03 | 0.00/10 | 12.520 | 0.360 | b | B | |  | 0.03/10 | 0.347 | 0.035 | ef | E | |  | 0.05/10 | 0.000 | 0.000 | f | E | |  | 0.1/10 | 0.000 | 0.000 | f | E | |  | 0.2/10 | 0.000 | 0.000 | f | E | | A19 | 0.03/10 | 0.197 | 0.025 | ef | E | |  | 0.05/10 | 0.427 | 0.081 | e | E | |  | 0.1/10 | 0.000 | 0.000 | f | E | |  | 0.2/10 | 0.000 | 0.000 | f | E |   **SUPPLEMENTAL TABLE 4｜The number of embryos for rooting culture and survived plantlets of five Chinese cabbage genotypes treated with different concentrations of EMS for different durations.** | | | | | | | | | | | | | | | |
| --- | --- | --- | --- | --- | --- | --- | --- | --- | --- | --- | --- | --- | --- | --- | --- |
| **EMS concentration (%)/time (min)** |  | **85-1** | |  | **12-2** | |  | **12-7** | |  | **A03** | |  | **A19** | |
|  |  | **Plantlet number** | **Embryo number** |  | **Plantlet number** | **Embryo number** |  | **Plantlet number** | **Embryo number** |  | **Plantlet number** | **Embryo number** |  | **Plantlet number** | **Embryo number** |
| 0.00/5 |  | 1617 | 2015 |  | － | － |  | － | － |  | 1377 | 1821 |  | － | － |
| 0.00/10 |  | 1709 | 2147 |  | － | － |  | － | － |  | 1218 | 1571 |  | － | － |
| 0.00/15 |  | 1540 | 1884 |  | － | － |  | － | － |  | 1332 | 1633 |  | － | － |
| 0.03/5 |  | 257 | 815 |  | 186 | 200 |  | 68 | 125 |  | 96 | 342 |  | 33 | 471 |
| 0.03/10 |  | 190 | 631 |  | 0 | 0 |  | 6 | 15 |  | 4 | 57 |  | 0 | 30 |
| 0.05/5 |  | － | － |  | 29 | 407 |  | 77 | 101 |  | 156 | 1009 |  | 0 | 177 |
| 0.05/10 |  | － | － |  | 5 | 136 |  | 0 | 0 |  | 0 | 0 |  | 18 | 54 |
| 0.05/15 |  | － | － |  | 0 | 0 |  | 0 | 0 |  | 0 | 11 |  | 0 | 0 |
| 0.10/5 |  | 424 | 1369 |  | 172 | 379 |  | 182 | 266 |  | 130 | 990 |  | 0 | 79 |
| 0.10/10 |  | 325 | 431 |  | 2 | 89 |  | 8 | 161 |  | 0 | 0 |  | 0 | 0 |
| 0.10/15 |  | 14 | 80 |  | 0 | 0 |  | 0 | 0 |  | 0 | 0 |  | 0 | 0 |
| 0.20/5 |  | 0 | 0 |  | 0 | 0 |  | 0 | 0 |  | 0 | 0 |  | 0 | 0 |
| 0.20/10 |  | 0 | 0 |  | 0 | 0 |  | 0 | 0 |  | 0 | 0 |  | 0 | 0 |
| 0.20/15 |  | 0 | 0 |  | 0 | 0 |  | 0 | 0 |  | 0 | 0 |  | 0 | 0 |

‘－’means no data.

**SUPPLEMENTAL TABLE 5｜Significant test for Impact of EMS on the Seedling Rate of Microspores**

**A Significant test for different time and genotypes under same EMS concentration treatment**

| Genotypes | | EMS concentration(%) / time (min) | Mean | standard deviation | 5% significant level | 1% significant level |
| --- | --- | --- | --- | --- | --- | --- |
| 85-1 | 0.00/5 | | 0.800 | 0.053 | a | A |
|  | 0.00/10 | | 0.797 | 0.032 | a | A |
|  | 0.00/15 | | 0.817 | 0.015 | a | A |
| A03 | 0.00/5 | | 0.753 | 0.035 | a | A |
|  | 0.00/10 | | 0.780 | 0.080 | a | A |
|  | 0.00/15 | | 0.813 | 0.012 | a | A |

| Genotypes | EMS concentration(%) / time (min) | Mean | standard deviation | 5% significant level | 1% significant level |
| --- | --- | --- | --- | --- | --- |
| 85-1 | 0.03/5 | 0.317 | 0.006 | cd | C |
|  | 0.03/10 | 0.303 | 0.049 | cd | C |
| 12-2 | 0.03/5 | 0.933 | 0.021 | a | A |
| 12-7 | 0.03/5 | 0.543 | 0.050 | b | B |
|  | 0.03/10 | 0.383 | 0.126 | c | C |
| A03 | 0.03/5 | 0.280 | 0.040 | d | C |
|  | 0.03/10 | 0.067 | 0.021 | e | D |
| A19 | 0.03/5 | 0.070 | 0.020 | e | D |
|  | 0.03/10 | 0.000 | 0.000 | e | D |

| Genotypes | EMS concentration(%) / time (min) | Mean | standard deviation | 5% significant level | 1% significant level |
| --- | --- | --- | --- | --- | --- |
| 12-2 | 0.05/5 | 0.073 | 0.023 | d | CD |
| 12-7 | 0.05/5 | 0.760 | 0.000 | a | A |
|  | 0.05/10 | 0.000 | 0.000 | d | D |
|  | 0.05/15 | 0.000 | 0.000 | d | D |
| A03 | 0.05/5 | 0.153 | 0.006 | c | C |
|  | 0.05/10 | 0.000 | 0.000 | d | D |
|  | 0.05/15 | 0.000 | 0.000 | d | D |
| A19 | 0.05/5 | 0.000 | 0.000 | d | D |
|  | 0.05/10 | 0.343 | 0.136 | b | B |
|  | 0.05/15 | 0.000 | 0.000 | d | D |

| Genotypes | EMS concentration(%) / time (min) | Mean | standard deviation | 5% significant level | 1% significant level |
| --- | --- | --- | --- | --- | --- |
| 85-1 | 0.1/5 | 0.310 | 0.010 | d | D |
|  | 0.1/10 | 0.750 | 0.061 | a | A |
|  | 0.1/15 | 0.173 | 0.006 | e | E |
| 12-2 | 0.1/5 | 0.453 | 0.038 | c | C |
|  | 0.1/10 | 0.020 | 0.017 | gh | F |
|  | 0.1/15 | 0.000 | 0.000 | h | F |
| 12-7 | 0.1/5 | 0.683 | 0.015 | b | B |
|  | 0.1/10 | 0.050 | 0.010 | g | F |
|  | 0.1/15 | 0.000 | 0.000 | h | F |
| A03 | 0.1/5 | 0.130 | 0.020 | f | E |
|  | 0.1/10 | 0.000 | 0.000 | h | F |
|  | 0.1/15 | 0.000 | 0.000 | h | F |
| A19 | 0.1/5 | 0.000 | 0.000 | h | F |
|  | 0.1/10 | 0.000 | 0.000 | h | F |
|  | 0.1/15 | 0.000 | 0.000 | h | F |

**B Significant test for different concentration and genotypes under same time treatment**

| Genotypes | EMS concentration(%) / time (min) | Mean | standard deviation | 5% significant level | 1% significant level |
| --- | --- | --- | --- | --- | --- |
| 85-1 | 0.00/5 | 0.800 | 0.053 | b | B |
|  | 0.03/5 | 0.317 | 0.006 | g | F |
|  | 0.1/5 | 0.310 | 0.010 | g | F |
|  | 0.2/5 | 0.000 | 0.000 | j | I |
| 12-2 | 0.03/5 | 0.933 | 0.021 | a | A |
|  | 0.05/5 | 0.073 | 0.023 | i | H |
|  | 0.1/5 | 0.453 | 0.038 | f | E |
|  | 0.2/5 | 0.000 | 0.000 | j | I |
| 12-7 | 0.03/5 | 0.543 | 0.050 | e | D |
|  | 0.05/5 | 0.760 | 0.000 | c | B |
|  | 0.1/5 | 0.683 | 0.015 | d | C |
|  | 0.2/5 | 0.000 | 0.000 | j | I |
| A03 | 0.00/5 | 0.753 | 0.035 | c | B |
|  | 0.03/5 | 0.280 | 0.040 | g | F |
|  | 0.05/5 | 0.153 | 0.006 | h | G |
|  | 0.1/5 | 0.130 | 0.020 | h | G |
|  | 0.2/5 | 0.000 | 0.000 | j | I |
| A19 | 0.03/5 | 0.070 | 0.020 | i | H |
|  | 0.05/5 | 0.000 | 0.000 | j | I |
|  | 0.1/5 | 0.000 | 0.000 | j | I |
|  | 0.2/5 | 0.000 | 0.000 | j | I |

| Genotypes | EMS concentration(%) / time (min) | Mean | standard deviation | 5% significant level | 1% significant level |
| --- | --- | --- | --- | --- | --- |
| 85-1 | 0.00/15 | 0.817 | 0.015 | a | A |
|  | 0.1/15 | 0.173 | 0.006 | b | B |
|  | 0.2/15 | 0.000 | 0.000 | c | C |
| 12-2 | 0.1/15 | 0.000 | 0.000 | c | C |
|  | 0.2/15 | 0.000 | 0.000 | c | C |
| 12-7 | 0.05/15 | 0.000 | 0.000 | c | C |
|  | 0.1/15 | 0.000 | 0.000 | c | C |
|  | 0.2/15 | 0.000 | 0.000 | c | C |
| A03 | 0.00/15 | 0.813 | 0.012 | a | A |
|  | 0.05/15 | 0.000 | 0.000 | c | C |
|  | 0.1/15 | 0.000 | 0.000 | c | C |
|  | 0.2/15 | 0.000 | 0.000 | c | C |
| A19 | 0.05/15 | 0.000 | 0.000 | c | C |
|  | 0.1/15 | 0.000 | 0.000 | c | C |
|  | 0.2/15 | 0.000 | 0.000 | c | C |

| Genotypes | EMS concentration(%) / time (min) | Mean | standard deviation | 5% significant level | 1% significant level |
| --- | --- | --- | --- | --- | --- |
| 85-1 | 0.00/10 | 0.797 | 0.032 | a | A |
|  | 0.03/10 | 0.303 | 0.049 | b | B |
|  | 0.1/10 | 0.750 | 0.061 | a | A |
|  | 0.2/10 | 0.000 | 0.000 | c | C |
| 12-2 | 0.1/10 | 0.020 | 0.017 | c | C |
|  | 0.2/10 | 0.000 | 0.000 | c | C |
| 12-7 | 0.03/10 | 0.383 | 0.126 | b | B |
|  | 0.05/10 | 0.000 | 0.000 | c | C |
|  | 0.1/10 | 0.050 | 0.010 | c | C |
|  | 0.2/10 | 0.000 | 0.000 | c | C |
| A03 | 0.00/10 | 0.780 | 0.080 | a | A |
|  | 0.03/10 | 0.067 | 0.021 | c | C |
|  | 0.05/10 | 0.000 | 0.000 | c | C |
|  | 0.1/10 | 0.000 | 0.000 | c | C |
|  | 0.2/10 | 0.000 | 0.000 | c | C |
| A19 | 0.03/10 | 0.000 | 0.000 | c | C |
|  | 0.05/10 | 0.343 | 0.136 | b | B |
|  | 0.1/10 | 0.000 | 0.000 | c | C |
|  | 0.2/10 | 0.000 | 0.000 | c | C |
